# Herbicide stress-induced DNA methylation changes in two *Zea mays* inbred lines differing in Roundup® resistance

**DOI:** 10.1007/s13353-021-00609-4

**Published:** 2021-01-29

**Authors:** Agata Tyczewska, Joanna Gracz-Bernaciak, Jakub Szymkowiak, Tomasz Twardowski

**Affiliations:** Institute of Bioorganic Chemistry Polish Academy of Sciences, Poznań, Poland

**Keywords:** DNA methylation, *Zea mays*, Crops, Herbicide, Abiotic stress, Adaptation

## Abstract

DNA methylation plays a crucial role in the regulation of gene expression, activity of transposable elements, defense against foreign DNA, and inheritance of specific gene expression patterns. The link between stress exposure and sequence-specific changes in DNA methylation was hypothetical until it was shown that stresses can induce changes in the gene expression through hypomethylation or hypermethylation of DNA. To detect changes in DNA methylation under herbicide stress in two local *Zea mays* inbred lines exhibiting differential susceptibility to Roundup®, the methylation-sensitive amplified polymorphism (MSAP) technique was used. The overall DNA methylation levels were determined at approximately 60% for both tested lines. The most significant changes were observed for the more sensitive *Z. mays* line, where 6 h after the herbicide application, a large increase in the level of DNA methylation (attributed to the increase in fully methylated bands (18.65%)) was noted. DNA sequencing revealed that changes in DNA methylation profiles occurred in genes encoding heat shock proteins, membrane proteins, transporters, kinases, lipases, methyltransferases, zinc-finger proteins, cytochromes, and transposons. Herbicide stress-induced changes depended on the *Z. mays* variety, and the large increase in DNA methylation level in the sensitive line resulted in a lower ability to cope with stress conditions.

**Supplementary Information:**

The online version contains supplementary material available at 10.1007/s13353-021-00609-4.

## Introduction

Herbicides are chemicals used to decrease weed population in crop fields, to protect crops and increase their yield, and to enhance harvest and processing efficiency of the agro-food industry. The most popular weedkillers are non-selective, i.e., they affect not only weed populations but also all plants growing in the sprayed area. Importantly, in the past few years, the amount and diversity of pesticides used in agriculture and horticulture have greatly increased, for example, in 2015, 4.1 Mt of herbicides were applied globally, which constitutes an increase of 35% compared to that used in 2001. By the year 2050, because of the rapid increase in human population and the expected land conversion into arable production, global pesticide applications are likely to increase further (Maggi et al. 2019).

Glyphosate (*N*-(phosphonomethyl)glycine) is an active compound of Roundup® (a highly effective, broad-spectrum, non-selective herbicide) and many other commercially available herbicides, that targets 5-enolpyruvylshikimate-3-phosphate synthase (EPSPS), an enzyme in the shikimate pathway that mediates the biosynthesis of aromatic amino acids in plants, bacteria, and fungi (Kanissery et al. 2019; Mertens et al. 2018). EPSPS of all higher plants appears to be inhibited by glyphosate, thus making it a non-selective herbicide that is active on a very wide range of plant species. Nevertheless, it remains unclear how glyphosate-induced inhibition of the shikimate pathway actually kills plants (Gomes et al. 2014). Some differences in the effects of glyphosate between plant species and biotypes are consistent with the observed differential susceptibility (Fuchs et al. 2002). It is assumed that insufficient aromatic amino acid production to maintain protein synthesis is the primary effect, and this is consistent with the slow development of symptoms (Duke and Powles 2008). Another possible cause of death, observed in velvetleaf tissues, is the restriction of water availability to the shoots, which is induced by lethal disruption of root processes. Moreover, because of interference with chlorophyll synthesis and its photodestruction caused by glyphosate-induced loss of carotenoids, a gradual inhibition of photosynthesis was also observed (Fuchs et al. 2002; Gomes et al. 2014). There is also evidence that the increased carbon flow to the shikimate pathway (by deregulation of the pathway due to EPSPS inhibition) results in shortages of carbon for other essential pathways (Duke and Powles 2008).

DNA methylation, one of the most important forms of epigenetic modification, has been shown to be involved in gene silencing at both transcriptional and post-transcriptional levels (Gallego-Bartolomé 2020; Agarwal et al. 2020). It plays significant roles in the regulation of gene expression (Meng et al. 2016), plant growth and development (Yang et al. 2014; Han et al. 2019; Agarwal et al. 2020), abiotic and biotic stress responses (Alonso et al. 2019; Kumar et al. 2020; Eriksson et al. 2020; Agarwal et al. 2020), environmental adaptation (Varotto et al. 2020; Gáspár et al. 2019), activity of transposable elements (Dowen et al. 2012; Garg et al. 2015; Lyu et al. 2017; Wang et al. 2018), defense against foreign DNA, and inheritance of specific gene expression patterns (Paszkowski and Whitham 2001; Matzke et al. 2009). Transcriptional gene silencing is associated with hypermethylation of promoter sequences, while post-transcriptional gene silencing is linked with hypermethylation of transcribed or coding sequences (Chinnusamy and Zhu 2009). In the plant genome, methylated cytosine residues (m^5^C) are found in three nucleotide-sequence contexts: symmetrical CG and two non-CG sites, namely symmetrical CNG and asymmetrical CNN sites (where N is A, T, or C). Moreover, different sequences have different genetic requirements for de novo or maintenance methylation.

The regulation of genomic methylation in plants is complex. Multiple DNA methyltransferases cooperate to establish and maintain methylation in a manner that reflects local sequence features (Matzke et al. 2009; Zhang et al. 2018; Bräutigam and Cronk 2018; Agarwal et al. 2020). Three distinct classes of enzymes are responsible for cytosine methylation. De novo methyltransferases DRM1 and DRM2 (domains rearranged methylase 1 and 2) catalyze new cytosine methylation, while the maintenance of symmetric CG and CHG methylation is mediated by the DNMT1-like enzyme MET1 and the plant-specific enzyme chromomethylase 3 (CMT3), respectively (Matzke et al. 2009; Chinnusamy and Zhu 2009; Zhang et al. 2018; Bräutigam and Cronk 2018; Agarwal et al. 2020). Inhibition of de novo methylation or inability to maintain the parental imprint after DNA replication are two passive ways of a DNA methylation loss, which were shown in *met1* mutants (Boyko and Kovalchuk 2008). Apart from that, DNA methylation can be removed enzymatically by the repressor of silencing 1 (ROS1), DEMETER (DME), and DME-like (DML)—proteins that possess DNA glycosylase-lyase activity (Chinnusamy and Zhu 2009; Zhang et al. 2018; Bräutigam and Cronk 2018; Agarwal et al. 2020).

The link between stress exposure and sequence-specific changes in DNA methylation was hypothetical until it was shown that stresses can induce changes in gene expression through hypomethylation or hypermethylation of DNA (Chinnusamy and Zhu 2009). In *Zea mays*, an immense loss of DNA methylation was reported under nitrogen deficiency; however, phosphate starvation led to much less changes (Mager and Ludewig 2018). It was also reported that in *Z. mays* under salinity stress, thousands of genes involved in cellular processes, metabolic processes, and signal transduction were associated with differential DNA methylation (Sun et al. 2018).

Cereals occupy the main position in the composition of a human diet, with rice, wheat, and *Z. mays* being the major staple cereals, having a share in excess of 70% among all food grains (Tyczewska et al. 2018; OECD-FAO Agricultural Outlook 2018–2027). Because of its high productivity, the importance for food and feed production and numerous industrial applications *Z. mays*, a plant originating from southern Mexico (Yang et al. 2019), is currently cultivated worldwide on a large scale (Langner et al. 2019; Handral et al. 2017). When grown under non-optimal conditions, *Z. mays* is exposed to environmental stresses, and hence, to minimize the influence of stress on the yield, it is essential to minimize the competition for water, light, and minerals that occurs between *Z. mays* seedlings and weeds growing in the field (Craine and Dybzinski 2013). The application of herbicides is the easiest and most widely used approach to eradicate weeds (Heap and Duke 2018).

Our aim was to identify the changes in DNA methylation levels and patterns underlying the resistance of *Z. mays* to the herbicide stress conditions. The two tested lines are inbred lines developed in Poland that naturally differ in susceptibility to Roundup®. In order to observe and analyze the changes in DNA methylation patterns, the methylation-sensitive amplification polymorphism (MSAP) technique was used.

## Materials and methods

### Plant material

*Z. mays* lines were chosen based on the results of field tests conducted to verify the response of 25 *Z. mays* inbred lines to Roundup® stress (K. Adamczewski, data not published). Based on these tests, we chose the S79757 line (sensitive to Roundup®, SL) as it showed the most prominent response to herbicide stress and the S245 line (tolerant to Roundup®, TL) as one of the most resistant (Supplementary Figure 1 in Mahmoud et al. (2020)). When the highest concentration of herbicide (Roundup® 360 SL) was used (300 g, 1.0 l/ha), the level of injuries to the TL variety was 40%, while it reached 85% in the SL 3 weeks after the application of the herbicide (rating is done based on phenotypic analyses of leaf chlorosis and wilting, inhibition of plant growth) (K. Adamczewski, data not published). Seeds from both lines were obtained from a local breeder (HR Smolice, Poland). The same two lines were used in the analyses described in Mahmoud et al. (2020) and Żywicki et al. (2015).

The seedlings were grown in a greenhouse in controlled temperature (22^o^C), humidity, and light conditions (16 h/8 h—light/dark) (Mahmoud et al. 2020; Żywicki et al. 2015). Uniform seedlings from both lines were selected and divided into two groups: one was sprayed with the herbicide Roundup® (1.0 l/ha, 300 g glyphosate) with adjuvant AS 500 SL (4.0 l/ha) 2 weeks after plants’ emergence (at the stage of 4–5 leaves), and the other (control) group was cultured without the herbicide treatment. To elucidate both early and late responses to stress conditions, for each tested sample, leaves were harvested from 6 plants at specified time points (6 h and 7 days after Roundup® application); leaves were harvested at the same time points for both tested plant groups. After collecting the plant material, the samples were immediately frozen in liquid nitrogen and stored at − 80 °C. Genomic DNA was isolated using the DNeasy Plant Maxi Kit (Qiagen) according to the manufacturer’s protocol. Before the DNA isolation, the plant material, pooled from 6 plants for each sample, was grind to a fine powder using TissueLyser II (Qiagen).

### DNA methylation analyses

#### Methylation-sensitive amplification polymorphism

The protocol was adapted from Yu et al. (2008) with some modifications. It consists of three major steps: digestion and ligation reactions, preamplification and amplification reactions, and detection. The designed adapters and primers for *EcoRI* and *HpaII*-*MspI* are described in Yu et al. (2008) and Xiong et al. (1999) and listed in Table 1.

**Table 1:** Adapter and primer sequences

Type of primer	*EcoRI* (E) (5′-3′)	*HpaII*/*MspI* (HM) (5′-3′)
*Adapter 1*	CTCGTAGACTGCGTACC (1E)	GACGATGAGTCCTGAG (1HM)
*Adapter 2*	AATTGGTACGCAGTC (2E)	CGCTCAGGACTCAT (2HM)
*PreAmp primer*	GACTGCGTACCAATTC (E00)	GATGAGTCCTGAGCGGC (HM00)
*Selective primers*	E00+AAC (E32)	HM00+CAA (HM1)
	E00+AAG (E33)	HM00+CAC (HM2)
	E00+ACC (E36)	HM00+CAG (HM3)
	E00+ACG (E37)	HM00+TAA (HM4)
	E00+AGC (E40)	HM00+TCC (HM5)
	E00+AGG (E41)	

##### Digestion and ligation reactions

DNA samples (300 ng) were separately digested using *EcoRI*-*HpaII* and *EcoRI*-*MspI* restriction enzyme pairs (60 U each, New England Biolabs) at 37 °C in an appropriate reaction buffer. DNA samples were purified by phenol/chloroform and precipitated with 3 M ammonium acetate pH 5.3, glycogen, and ethanol overnight at − 20 °C.

The total amount of digested DNA was used for the ligation reactions with 5 pmoles of 1E and 2E adapters and 50 pmoles of 1HM and 2HM adapters. The ligation was performed in 25 μl of total volume with 2 μl of T4 DNA ligase (10U, Fermentas) for 1 h at 22 °C.

##### Preamplification and selective amplification

Preamplification PCR reactions were performed in a final volume of 50 μl containing 5 μl of ligation products, 75 ng of E00 and HM00 PreAmp primers (Table 1), and Dream Taq (5 U/μl, Fermentas). DNA fragments were amplified for 25 cycles of 94 °C for 90 s, 56 °C for 30 s, and 72 °C for 1 min, prior to selective amplification. The PCR products were diluted 1:25 (v:v) with ddH_2_O. Five microliters of the diluted preamplified PCR products was used for selective PCR reactions with Dream Taq (5 U/μl) and 40 ng of a selective primer pair E00+3/HM00+3. Selective primers have additional 3 nucleotides at their 3′ ends (Table 1). The reactions were performed in a total volume of 50 μl. The PCR conditions were as follows: 13 cycles at 94 °C for 30 s, 65^o^C for 30 s (reduced by 0.7 °C at each cycle), and 72 °C for 1 min followed by 23 cycles: 94 °C for 30 s, 56 °C for 30 s, and 72 °C for 1 min.

##### Detection assay

The total amount of DNA selective amplification products was mixed with a loading buffer (6×), heated for 5 min at 95 °C, and rapidly chilled on ice. The entire mixtures were loaded on 6% denaturing polyacrylamide gels (50 cm × 33 cm, one for each primer combination). Electrophoresis was performed at a constant power of 700 V for 18 h with cooling with *GeneRuler 1kb DNA ladder* (Fermentas). The detection of end products was performed using Sybr Gold (Thermo Fisher Scientific).

### Isolation, cloning, and sequencing of MSAP fragments

Differentiating DNA fragments were removed from the polyacrylamide gels under UV light by using a razor blade. The fragments were eluted from the gel in 200 μl of 0.3 M ammonium acetate and incubated overnight at 4 °C under shaking. DNA samples were purified by phenol/chloroform and precipitated with 3 M ammonium acetate pH 5.3, glycogen, and ethanol overnight at − 20 °C; the samples were then centrifuged for 30 min at 4 °C, dried, and resuspended in ddH_2_O. Aliquots (5 μl) were used for reamplification. PCR reactions were performed using the same primer combinations and reaction conditions as those used in selective amplification. The PCR products were then ligated into a vector pCR™2.1-TOPO using a ligation TOPO TA Cloning Kit (Life Technologies). Ligation was performed for 30 min at room temperature in a final volume of 6 μl using 10 ng of a vector and 30 ng of DNA. The ligation products were transformed into an *Escherichia coli* DH5α strain. The detection of end products was based on the blue/white screening test. Colony PCR was performed for samples taken from white colonies using Clone ID Colony PCR Master Mix (Lucigen) and 12 pmoles each of universal M13 Forward and M13 Reverse primers (Genomed). The PCR conditions were as follows: initial denaturation at 98 °C for 2 min, 29 cycles of 98 °C for 30 s, 54 °C for 30 s, and 72 °C for 3 min, followed by complementary elongation at 72 °C for 10 min. The quality of PCR products was checked by agarose electrophoresis in 1% gel along with *GeneRuler 100 bp Plus DNA Ladder* (Fermentas). Only full-length DNA samples were sequenced using the Sanger sequencing method at Genomed.

### Bioinformatics analysis

The obtained DNA sequences were analyzed using the Basic Local Alignment Search Tool (BLASTn, NCBI, https://blast.ncbi.nlm.nih.gov/Blast.cgi) and *Z. mays* GDB (B73 filtered gene sets 4a.53 for RefGen_v1, https://www.maizegdb.org/). GO functional classification was performed using Blast2GO (https://www.blast2go.com/) at the default settings. The sequences were blasted using the NCBI Blast service (QBlast) and blastx-fast program. Blast expectation value (*E* value) was set at 1.0E−3. Next, the GO ontology mapping and InterProScan were performed. GO mapping was performed against extensively curated Gene Ontology annotated proteins, to obtain functional labels. The used data originates from the Gene Ontology Association and Uniprot ID-Mapping. The public EMBL-EBI InterPro web-service was used to scan sequences against InterPro’s signatures at the default settings. GO annotation was performed with Annotation CutOff set at 55, and *E* value-hit-filter set at 1.0E−6. Enrichment analysis was performed using Fisher’s exact test.

### Methylation analysis of CentC in *Z. mays*

Total *Z. mays* DNA was isolated using a DNeasy Plant Maxi Kit (Qiagen) according to the manufacturer’s instructions. Target CentC methylation was analyzed with methylation-sensitive restriction enzymes (*HpaII* and *MspI*, 60U, New England Biolabs) and Southern blot hybridization, according to previously published protocols (Mette et al. 2000; Aufsatz et al. 2002). A ^32^P-labeled RNA probe (5′-ATGAGTTTTGGACCTAAAGTAGTGGATTGG-3′) was used for probing the Southern blots.

## Results

### Extent and pattern of global DNA methylation under control condition and herbicidal stress condition in *Z. mays*

**Fig. 1:**
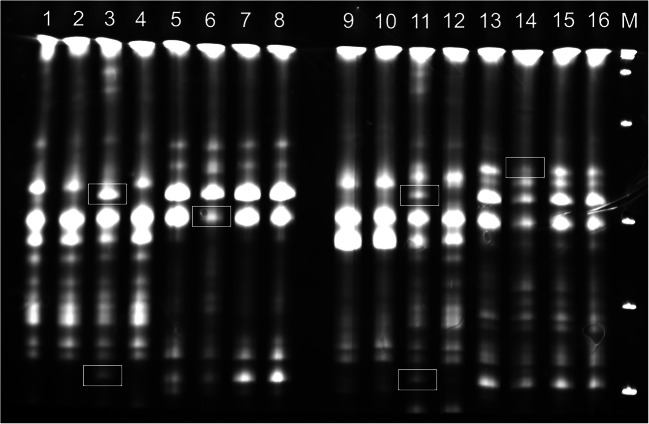
Electrophoregram of DNA samples digested with* HpaI *and* MspII*, amplified using E41-HM5 primers. 1–4 and 9–12—TL line; 5–8 and13–16—SL line; 1–8—6 h after herbicide application; 9–16—7 days after the application of herbicide; 1, 3, 5, 7, 9, 11, 13, and 15 (odd numbers)—control samples; 2, 4, 6, 8, 10, 12, 14, and 16 (even numbers)—herbicide-treated samples. M, *GeneRuler 1kb DNA ladder 250*–*10,000 bp* (Thermo Fisher Scientific). Some of the differences in the MSAP profiles between samples are marked with white boxes

To evaluate DNA cytosine methylation at 5′-CCGG-3′ sequences in two *Z. mays* lines that are either tolerant (S245, TL) or sensitive (S79757, SL) to herbicidal stress, 24 primer pairs were used. The enzymes used in the digestion of DNA are sensitive to methylation at either CG and CNG (*HpaII*: ^m^C^m^CGG) or only CNG (*MspI*: ^m^CCGG) sites. To elucidate early and late responses to herbicide stress, the samples were collected 6 h and 7 days after Roundup® treatment. A total of 888 and 826 clear bands were amplified from TL and SL, respectively. For HpaI and MspII restriction enzymes, four distinct restriction patterns can be distinguished (Table 2). A sample photo showing the results of *Z. mays* DNA amplification for the E41-HM5 primer pair is given in Fig. 1. As can be seen, a highly repetitive banding pattern can be observed for the amplification products for TL (lines 1–4, 9–12) and SL samples (lines 5–8,13–16). The differences in the amplification profiles between the two tested *Z. mays* lines (TL and SL) are also clearly visible. Additionally, differential bands attributed to the changes in DNA methylation between samples derived from TL or SL under herbicide stress are markedly noticeable (change in intensity, appearance, or disappearance of bands, marked by boxes). The biggest differences in the banding pattern were observed for SL sample 6 h after the application of a herbicide, compared to the control (line 6).

**Table 2:** Restriction patterns for *HpaII*/*MspI* restriction enzymes. The numbers of bands corresponding to a particular restriction pattern in all tested lines and conditions are given

Sample ID	Restriction enzyme digestion (*HpaII*/*MspI*)			
	(+/+)	(+/−)	(−/+)	(−/−)
TL6h-C/TL7d-C	330/320	178/211	172/153	208/204
TL6h-H/TL7d-H	325/360	225/178	136/141	202/209
SL6h-C/SL7d-C	348/194	164/164	120/227	194/241
SL6h-H/SL7d-H	323/322	203/165	143/136	157/203

TL6h-C—TL line 6 h after spraying, control; TL6h-H—TL line 6 h after spraying, herbicide; TL7d-C—TL line 7 days after spraying, control; TL7d-H—TL line 7 days after spraying, herbicide; SL6h-C—SL line 6 h after spraying, control; SL6h-H—SL line 6 h after spraying, herbicide; SL7d-C—SL line 7 days after spraying, control; SL7d-H—TL line 7 days after spraying, herbicide. Restriction enzyme digestion pattern is given in brackets and the numbers of corresponding bands are given below the patterns

**Fig. 2:**
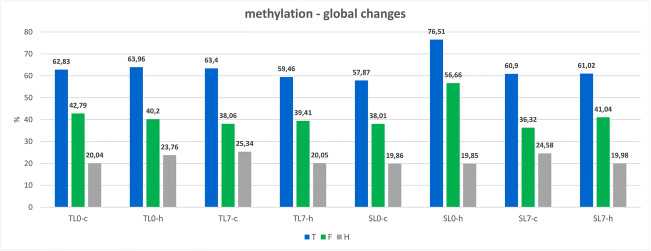
Global DNA methylation changes in TL and SL *Z. mays* lines resulting from herbicidal stress conditions. T, total methylation level; F, fully methylates sites; H, hemimethylated sites. TL6h-C, TL line 6 h after spraying, control; TL6h-H, TL line 6 h after spraying, herbicide; TL7d-C, TL line 7 days after spraying, control; TL7d-H, TL line 7 days after spraying, herbicide; SL6h-C, SL line 6 h after spraying, control; SL6h-H, SL line 6 h after spraying, herbicide; SL7d-C, SL line 7 days after spraying, control; SL7d-H, TL line 7 days after spraying, herbicide. The biggest changes in DNA methylation in two tested *Z. mays* lines under herbicide stress conditions were observed in SL line 6 h after herbicide application. These changes are attributed to a large increase (by 18.65%) in the number of fully methylated sites

Under the control conditions, the total methylation level of 5′-CCGG-3′ sequences averaged between 63.11% in TL and 59.38% in SL. The extent of DNA methylation ranged from 62.38% (6 h after treatment) to 63.40% (7 days after treatment) in TL and from 57.87% (6 h after treatment) to 60.90% (7 days after treatment) in SL. Herbicidal stress increased the percentage of total methylated bands in both tested lines 6 h after Roundup® treatment. The change was very slight for the TL (1.13%) compared to SL (18.64%) (Fig. 2). Seven days after herbicide treatment, a decrease in the level of methylation was observed for the TL line (3.94%), and almost no difference was noted for the SL line (0.12%). Importantly, the fully methylated loci were always more abundant than the hemi-methylated ones.

For TL, a decrease in fully methylated bands (2.59%) and an increase in hemi-methylated bands (3.72%) 6 h after the treatment were observed, while 7 days after treatment, there was a slight increase in fully methylated bands (1.35%) and a decrease in hemi-methylated bands (5.29%). The drastic increase in methylation level in SL line 6 h after herbicide treatment was attributed to the increase in fully methylated bands (18.65%), while the level of hemi-methylated bands stayed almost constant (0.01%). Seven days after treatment, as the total methylation level stayed unchanged in treated vs control plants, there was an increment in fully methylated bands (4.72%) and a decrease in hemi-methylated bands (4.60%) (Table 3, Fig. 2).

**Table 3:** The binding patterns revealed by the MSAP analysis: no change in methylation (A–D), demethylation (E–J), and methylation (K–P) events. Number of bands and percentages of occurrence are given for both tested *Z. mays* lines and all tested conditions

Cutting class	Restriction enzyme digestion pattern change (*HpaII/MspI*) → (*HpaII/MspI*)	*Z. mays* samples, percentage (no. of bands)			
		TL6h-C/TL6h-H	TL7d-C/TL7d-H	SL6h-C/SL6h-H	SL7d-C/SL7d-H
A (I to I)	(+/+) → (+/+)	30.29% (269)	29.73% (264)	19.49% (161)	34.02% (281)
B (II to II)	(+/−) → (+/−)	13.63% (121)	14.41% (128)	9.44% (78)	13.68% (113)
C (III to III)	(−/+) → (−/+)	13.06% (116)	7.99% (71)	7.99% (66)	10.17% (84)
D (IV to IV)	(−/−) → (−/−)	15.65% (139)	12.27% (109)	9.08% (75)	14.89% (123)
**Total**		**72.63% (645)**	**64.41% (572)**	**46% (380)**	**72.76% (601)**
E (II to I)	(+/−) → (+/+)	1.01% (9)	4.62% (41)	0.85% (7)	3.51% (29)
F (III to I)	(−/+) → (+/+)	3.94% (35)	3.94% (35)	1.21% (10)	2.78% (23)
G (IV to I)	(−/−) → (+/+)	0.79% (7)	2.59% (23)	1.94% (16)	0.60% (5)
H (III to II)	(−/+) → (+/−)	0.68% (6)	0.22% (2)	0.85% (7)	0.24% (2)
I (IV to II)	(−/−) → (+/−)	5.18% (46)	4.62% (41)	7.87% (65)	1.94% (16)
J (IV to III)	(−/−) → (−/+)	1.80% (16)	3.26% (29)	4.60% (38)	1.45% (12)
**Total**		**13.4% (119)**	**19.25% (171)**	**17.32% (143)**	**10.52% (87)**
K (I to II)	(+/+) → (+/−)	4.28% (38)	1.58% (14)	2.90% (24)	2.78% (23)
L (I to III)	(+/+) → (−/+)	1.91% (17)	2.03% (18)	11.5% (95)	3.03% (25)
M (II to III)	(+/−) → (−/+)	0.56% (5)	1.46% (13)	2.06% (17)	1.33% (11)
N (I to IV)	(+/+) → (−/−)	0.68% (6)	3.60% (32)	8.35% (69)	0.97% (8)
O (II to IV)	(+/−) → (−/−)	4.84% (43)	5.29% (47)	8.72% (72)	5.20% (43)
P (III to IV)	(−/+) → (−/−)	1.69% (15)	2.36% (21)	3.15% (26)	3.39% (28)
**Total**		**13.96% (124)**	**16.32% (145)**	**36.68% (303)**	**16.70% (138)**

TL6h-C—TL line 6 h after spraying, control; TL6h-H—TL line 6 h after spraying, herbicide TL7d-C—TL line 7 days after spraying, control; TL7d-H—TL line 7 days after spraying, herbicide; SL6h-C—SL line 6 h after spraying, control; SL6h-H—SL line 6 h after spraying, herbicide; SL7d-C—SL line 7 days after spraying, control; SL7d-H—TL line 7 days after spraying, herbicide. (+) Sample digested, (−) sample not digested with appropriate restriction enzyme—*HpaII* or *MspI*. Restriction enzyme digestion pattern change is indicated with an arrow “**→**”

### Herbicide-induced changes in the level of DNA methylation in *Z. mays* genotypes that differ in their tolerance to herbicidal stress

**Fig. 3:**
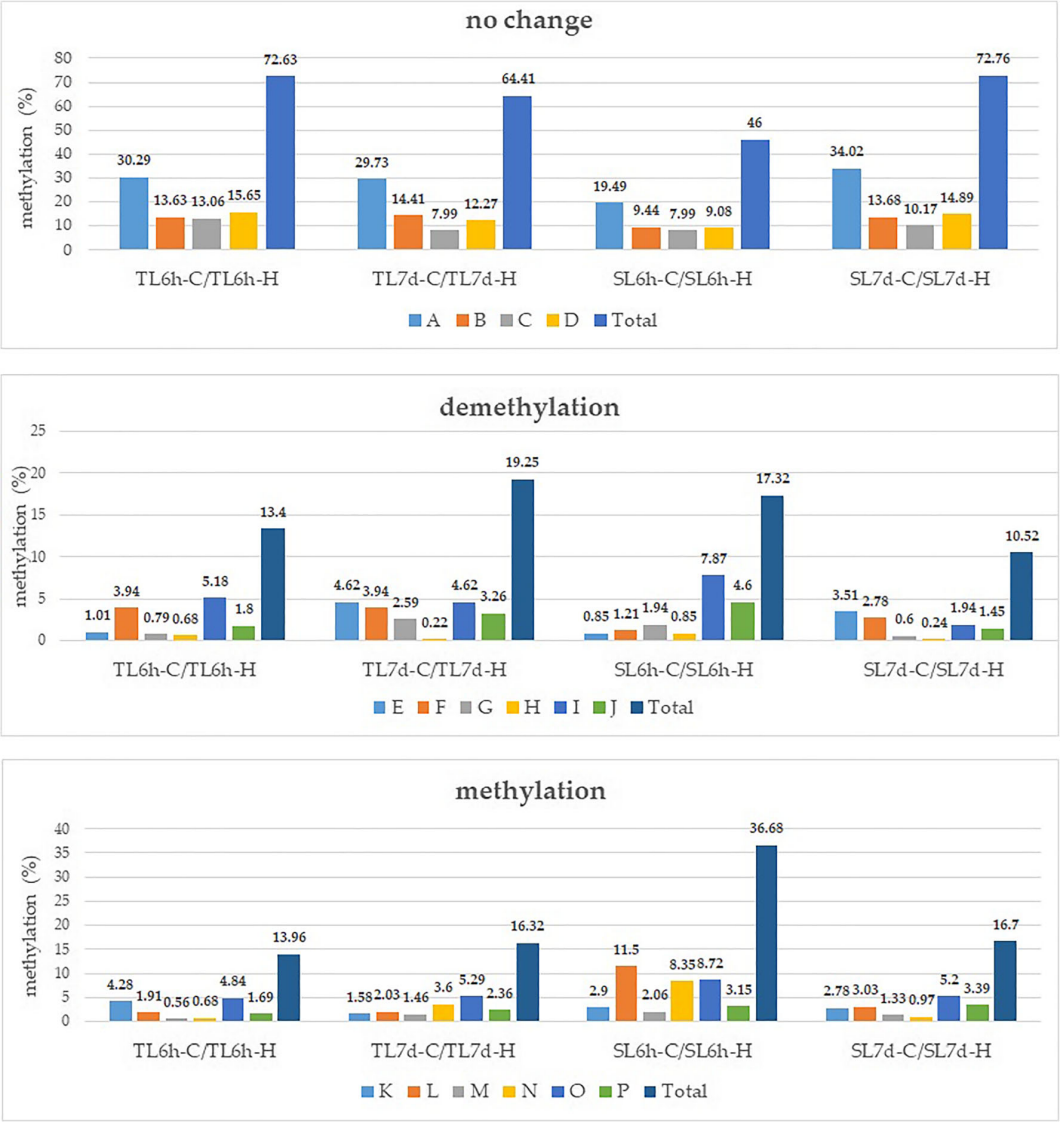
The binding patterns obtained from the MSAP analysis: no change in DNA methylation level (A–D), demethylation (E–J), and methylation (K–P) events in TL and SL *Z. mays* lines under herbicidal stress conditions, given in percentages. TL6h-C, TL line 6 h after spraying, control; TL6h-H, TL line 6 h after spraying, herbicide; TL7d-C, TL line 7 days after spraying, control; TL7d-H, TL line 7 days after spraying, herbicide; SL6h-C, SL line 6 h after spraying, control; SL6h-H, SL line 6 h after spraying, herbicide; SL7d-C, SL line 7 days after spraying, control; SL7d-H, TL line 7 days after spraying, herbicide

To analyze the changes in cytosine methylation patterns under herbicidal stress, all possible banding patterns between control and Roundup® stress in TL and SL lines were calculated and compared (Table 3, Fig. 3). The MSAP analysis revealed 16 banding patterns, and they represent no change in methylation level (A–D), demethylation (E–J), and methylation (K–P) events (Karan R’ DeLeon et al. 2012).

For TL, the methylation of 72.63% and 64.41% of 5′-CCGG-3′ sites remained unchanged under herbicide treatment, 6 h and 7 days after spraying, respectively. We observed a difference in demethylation events at two different time points in TL—13.40% vs 19.25%—and a difference in methylation events—13.96% and 16.32% at 6 h and 7 days after treatment, respectively. For SL, out of 826 bands, 46% and 72.76% of 5′-CCGG-3′ sites remained unchanged under herbicide treatment, 6 h and 7 days after spraying, respectively. There was a decrease in demethylation events at 7 days post-treatment as compared to that at 6 h after treatment (17.32% to 10.52%). The largest difference was observed for the methylation events (19.98%), and it decreased significantly from 36.68% to 16.70% 1 week after spraying. The test of independence between different methylation patterns (total methylation, full methylation, and hemi-methylation) for control and herbicide stress conditions was carried out using the chi-square test (Supplementary Table S1, Supplementary Table S2).

### Sequencing and GO analysis of chosen differentially methylated DNA fragments

Selected DNA fragments were isolated from polyacrylamide gels and sequenced at Genomed (Poland). A total of 197 DNA sequences were obtained and subjected to bioinformatics analysis; primer and vector sequences were removed prior to analysis. NCBI and MaizeGDB databases were used for sequence analysis. Of the 197 fragments of 100 to 1200 bp, as many as 151 fragments had sequences that matched with more than 90% accuracy in at least one of the databases searched (Supplementary Table S3). The matched sequences belonged not only to *Z. mays* but also to the related bicolor sorghum (*Sorghum bicolor*) and rice (*Oryza sativa*).

**Fig. 4:**
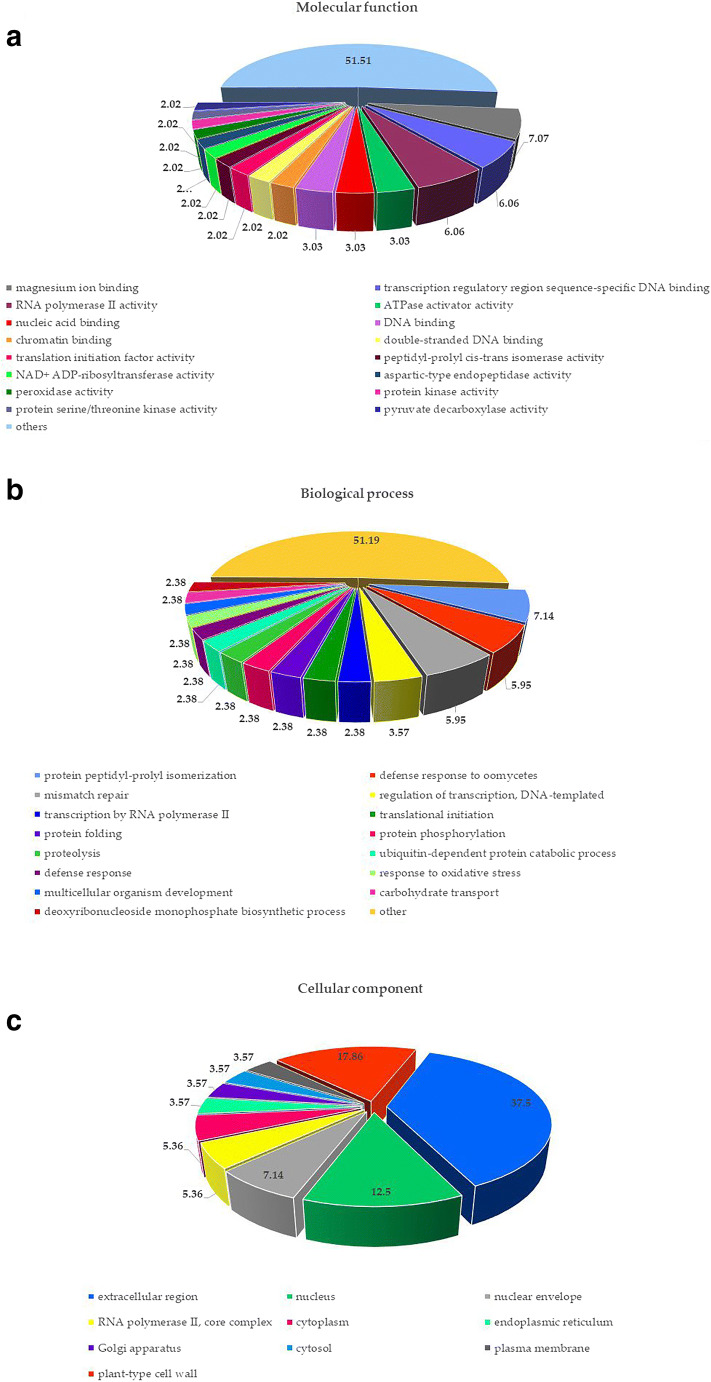
A graph showing the percentage of GO enrichment analysis in **a** “molecular function” category, **b** “biological process” category, and **c** “cellular component” category. The values are given in percentages (%)

GO functional classification was performed using Blast2GO (https://www.blast2go.com/) at the default settings. Gene Ontology terms were assigned to 87 target genes, which were described by 67 terms in “molecular function,” 58 terms in “biological process,” and 19 terms in “cellular compartment” categories (Supplementary Table S4). Highly represented terms in the “molecular function” category (Fig. 4a) included “magnesium ion binding” (GO:0000287), “transcription regulatory region sequence-specific DNA binding” (GO:0000976), and “RNA polymerase II activity”(GO:0001055); in the “biological process” category (Fig. 4b)—“defense response to oomycetes” (GO:0006468), “mismatch repair” (GO:0006298), and “regulation of transcription, DNA-templated” (GO:0006355); and in the “cellular compartment” category (Fig. 4c)—“extracellular region” (GO:0005576), “nucleus” (GO:0005634), and “nuclear envelope” (GO:0005635). Importantly, GO terms associated with the membranes in the “cellular compartment” category were “plasma membrane” (GO:0005886), “membrane” (GO:0016020), “integral component of membrane” (GO:0016021), “integral component of endoplasmic reticulum membrane” (GO:0030176), and “anchored component of membrane” (GO:0031225). Enrichment analysis (Fisher’s exact test) indicated “hydrolase activity” (GO:0016787) is significant at *p* value 0.05. Moreover, according to the KEGG analysis (Kanehisa and Goto 2000), 87 target genes were significantly enriched in 20 pathways including “drug metabolism - other enzymes,” “drug metabolism - cytochrome P450,” and “metabolism of xenobiotics by cytochrome P450” (Supplementary Table S4).

Among the identified sequences were genes coding for transferases, transporter proteins, methyltransferases, hydrolases, transposons, ribosomal proteins, cytochromes, and proteins participating in transcription processes and involved in stress responses (Supplementary Table S3).

### Methylation analysis of repetitive sequences

**Fig. 5:**
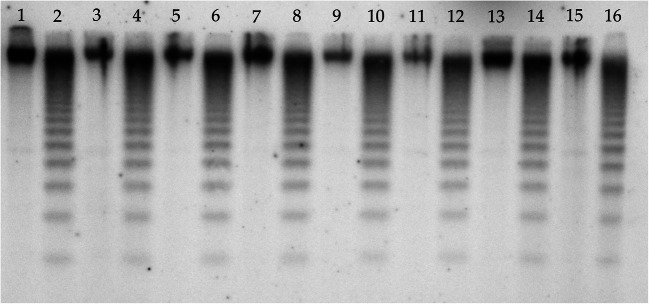
Southern blot analysis of the changes in cytosine DNA methylation in CentC repetitive sequences in *Z. mays* under herbicide stress conditions. 1, 3, 5, 7, 9, 11, 13, and 15 (odd numbers)—*HpaII *restriction enzyme digestion; 2, 4, 6, 8, 10, 12, 14, and16 (even numbers)—*MspI* restriction enzyme digestion; 1 and 2—TL6h-C; 3 and 4—TL6h-H; 5 and 6—TL7d-C; 7 and 8—TL7d-H; 9 and 10—SL6h-C; 11 and 12—SL6h-H; 13 and 14—SL7d-C; 15 and 16—SL7d-H

Southern blot analysis of the centromere-specific satellite repeat CentC (Fig. 5) was performed to analyze whether herbicidal stress influences DNA methylation in such highly methylated genomic regions. In the bioinformatic analysis of sequences with differential DNA methylation, centromeric fragments in *Z. mays* genomes were identified (Supplementary Table S3). However, it was observed that under herbicide stress conditions, the methylation pattern of the CentC did not change (Fig. 5). The digestion patterns reflected substantial methylation of CGs and non-CGs, as indicated by the increased cleavage with the methylation-insensitive isoschizomer (M) compared to that with the methylation-sensitive isoschizomer (H).

## Discussion

Environmental conditions may sometimes exert a detrimental effect on the development and maturation of organisms. This is particularly true for plants. Because of their sedentary lifestyle, plants require short-term strategies to rapidly and efficiently re-adapt their metabolism and thus have developed unique features in terms of habitat, growth, and reproduction (Boyko and Kovalchuk 2008). A comprehensive understanding of the mechanism of action of glyphosate-based herbicides is important as it affects the growth of plants not only by inhibiting EPSPS but also by altering several crucial plant physiological processes (e.g., photosynthesis, carbon metabolism, mineral nutrition, and oxidative events) (Geiger et al. 1986; Hirayama and Shinozaki 2010; Orcaray et al. 2012; Romero et al. 2011; Ghanizadeh and Harrington 2017; Gomes et al. 2014).

Several authors reported previously changes in DNA methylation in *Z. mays* and other plant species under abiotic stress conditions (Mager and Ludewig 2018; Sun et al. 2018; Kong et al. 2020; Wang et al. 2014; Wang et al. 2015; Uthup et al. 2011; Tan 2010). Massive loss of DNA methylation in CG and CNG contexts has been observed in *Z. mays* under nitrogen deficiency. In contrast, phosphorus deficiency caused only a slight change in DNA methylation level, mainly in the CG context (Mager and Ludewig 2018). Salt stress also influenced the DNA methylation level in *Z. mays*, and it has been concluded that highly methylated CpG islands might participate in the regulation of gene transcription under salt stress. Several other reports showed the influence of stress conditions on DNA methylation in wheat, barley, and rice (Kong et al. 2020; Wang et al. 2014; Wang et al. 2015; Uthup et al. 2011; Tan 2010).

Previously, it was shown that herbicidal stress influences miRNA expression (Żywicki et al. 2015) and that some of the traits related to herbicide resistance may be encoded in the genome (Mahmoud et al. 2020). Here, we focused our efforts on analyzing the influence of herbicidal stress on changes in DNA methylation in the same two *Z. mays* lines, which are differentially susceptible to the systemic herbicide Roundup®. The results of the analysis of global methylation changes showed a high DNA methylation level in control samples (approximately 63% and 58% in TL in SL, respectively) (Fig. 2). This observation is consistent with a previous report showing that *Z. mays* is characterized by an overall high level of DNA methylation (Li et al. 2014). This high rate of DNA methylation is attributed to the high content of transposons and repetitive sequences in *Z. mays* genomes as it has been estimated that the fraction of the genome that appears to be repetitive is 77%, which in general is randomly distributed in the genome (Meyers et al. 2001).

The application of Roundup® to *Z. mays* plants resulted in only slight changes in total DNA methylation levels in the TL line (Fig. 2)—an increase by 1.13% at 6 h and a decrease of 3.4% at 7 days after the treatment. In contrast, a large increase in the level of DNA methylation (attributed to a large increase in fully methylated context) of 18.64% was observed in the SL line at 6 h after herbicide treatment (Fig. 3, Table 2). Seven days after herbicide spraying, the level of DNA methylation in SL returned to the control level (61%) (Fig. 3, Table 3). The differences in the response to herbicide stress conditions between the tested inbred lines are clearly visible. In *Arabidopsis* plants subjected to glyphosate stress, 9205 differentially methylated regions have been identified across the genome (Kim et al. 2017). It was shown that the methylation patterns were dose-sensitive and, to a degree, stress-specific. Additionally, two out of seven genes in the shikimate pathway were differentially methylated as a result of the herbicide stress. Herein, we did not detect changes in the methylation pattern in genes encoding shikimate pathway enzymes; however, previous changes in the coding sequences of two shikimate pathways enzymes (bifunctional 3-dehydroquinate dehydratase/shikimate dehydrogenase and chorismate synthase) in the two tested *Z. mays* lines (TL and SL) were detected (Mahmoud et al. 2020).

Other genes linked with herbicide stress responses are encoding transporter proteins. According to GO analysis, the term highly represented in the “cellular compartment” category was “extracellular region” constituting 37.5%, and the categories associated with membranes constituted 12.5%. A group of transporter proteins (Supplementary Table S3) differentially methylated following herbicide application was identified among the sequences analyzed. Importantly, in rice subjected to atrazine (ATR) stress, and broad bean to glyphosate stress, among differentially methylated genes were those enriched in functions associated with transport activity (Lu et al. 2016; Denis and Delrot 1993). Similarly, enrichment in genes associated with phosphate has been reported in *Arabidopsis* subjected to glyphosate stress (Kim et al. 2017). Notably, out of 13 miRNAs differentially expressed in the two tested *Z. mays* lines following glyphosate application, miR444 and miR827 have been shown to regulate the phosphate transport pathways, which seem to be common for Pi and glyphosate uptake (Żywicki et al. 2015). Moreover, recently, single nucleotide polymophisms (SNPs) and indels were detected in genes encoding phosphate transporters in TL and SL *Z. mays* lines (Mahmoud et al. 2020). This is particularly important, since glyphosate has been shown to be recognized by phosphate transporters (Hetherington et al. 1998). Notably, phosphate transporters 1 and 2 were implicated in the active transport of glyphosate into plant cells (Mahmoud et al. 2020; Denis and Delrot 1993; Hetherington et al. 1998; Morin et al. 1997; Shaner 2009; Gomes et al. 2016).

Out of two types of herbicide resistance, non-target site resistance (NTSR) may occur via enhanced xenobiotics detoxification, herbicide metabolism, or translocation (Markus et al. 2018; González-Torralva et al. 2012). Xenobiotics detoxification is mediated by cytochrome P450 monooxygenases (P450s), glutathione transferases (GSTs), or ATP-dependent (ATP-binding cassette (ABC)) transporters in plants (Jensen and Møller 2010). Cytochrome P450 monooxygenases constitute a group of proteins reported as affected by herbicide stress (Lu et al. 2016; Markus et al. 2018; Jensen and Møller 2010; Samsel and Seneff 2013). Plant P450s are involved in acclimatization to biotic and abiotic stresses and were shown to mediate herbicide metabolism (Siminszky 2006). Moreover, P450s were shown to be involved in the degradation of ATR in rice (Rong Tan et al. 2015). These results are corroborated by KEGG analysis as the enriched pathways included “drug metabolism - other enzymes,” “drug metabolism - cytochrome P450,” and “metabolism of xenobiotics by cytochrome P450.”

The involvement of ABC transporters in glyphosate resistance was reported in *Conyza canadensis* (Tani et al. 2015; Tani et al. 2016; Moretti et al. 2017), *Conyza bonariensis,* and *C. canadensis* (Moretti et al. 2017). Herein, one of the DNA fragments with changed DNA methylation level was identified as the gene encoding the ATP binding protein belonging to the family of ABC transporters and another one as encoding multidrug and toxic compound extrusion (MATE) protein (Supplementary Table S3). ABC transporters are present in all living organisms (Jasinski et al. 2003; Lefèvre and Boutry 2018), and they constitute a very large and diverse family of proteins. The function of ABC transporters is the transport of various compounds across the cell membranes, including lipids, sugars, amino acids, proteins, secondary metabolites, heavy metal ions, and xenobiotics (including herbicides) (Tani et al. 2015). Previously, structural changes in genes encoding the MATE family of proteins have been demonstrated in *Z. mays* inbred lines differentially resistant to the glyphosate-based herbicide Roundup® (TL and SL) (Mahmoud et al. 2020). Both protein families, ABC and MATE transporters, play significant roles in the transport of xenobiotics and other small molecules and may contribute to glyphosate transport and distribution in plants (Markus et al. 2018). The differences in the DNA methylation level of the protein-coding fragment belonging to the ABC and MATE transporter family in the tested *Z. mays* lines may suggest their role in the increased adaptation of certain *Z. mays* lines to herbicidal stress conditions.

Environmental factors may influence DNA methylation either by directly inhibiting enzymes that catalyze DNA methylation or by changing the availability of substrates required for those enzymatic reactions such as the availability and utilization of methyl donor groups (Markus et al. 2018; Munksgaard et al. 1995; Meng et al. 2018). Recently, it was shown that in rice, in response to ATR herbicide, DNA methyltransferases, histone methyltransferases, and DNA demethylases were differentially expressed (Kim et al. 2017). Herein, several methyltransferases (among others SAM-dependent carboxyl methyltransferase, benzoate carboxyl methyltransferase, gamma-tocopherol methyltransferase) had altered the DNA methylation levels in response to glyphosate-based herbicide (Supplementary Table S3) suggesting their possible role in glyphosate-based herbicide stress resistance in *Z. mays*.

Global analysis of plants such as *Arabidopsis* and rice suggests that the majority of transposons are inactive, methylated, and targeted by siRNAs (Lisch 2008). Given the large number of potentially active elements in most eukaryotic genomes, global activation could result in an overwhelmingly high level of mutation. Nevertheless, McClintock suggested that all types of stresses could potentially reshape a plant genome via transposon activation (McClintock 1984), a hypothesis that has been confirmed by other authors (Kim et al. 2017; Negi et al. 2016; Galindo-González et al. 2018; Boyko and Kovalchuk 2008; Hashida et al. 2006). Previously, it was shown that transposable elements may contribute to the activation of *Z. mays* genes in response to abiotic stress (cold, heat, salinity, and UV) as some TE families were associated with stress-responsive expression of nearby genes, and some TE families may act as local enhancers of stress-responsive expression (Makarevitch et al. 2015). Four to nine different TE families were associated with the upregulation of gene expression in each of these stress conditions, thus affecting up to 20% of the genes upregulated in response to abiotic stress, and as many as 33% of the genes that are expressed only in response to stress (Makarevitch et al. 2015). Importantly, changes in the DNA methylation status of TEs have been linked with herbicide stress responses (Markus et al. 2018). Herein, changes in DNA methylation were detected, among others, in miniature inverted-repeat transposable element Hbr22, Mu transposon, retrotransposons, and a helitron (Supplementary Table S3).

## Conclusions

The selective pressure exerted by persistent application of herbicides may extort adaptive responses not only in weeds (a phenomenon observed for many years), but also in crop plants. The present analysis showed that herbicide stress, depending on the natural susceptibility of *Z. mays* varieties to herbicide, caused various changes in the DNA methylation levels and patterns in *Z. mays* varieties depending on their natural susceptibility to herbicides. This, in turn, may be reflected in the changes in the expression of particular genomic fragments that may result in a heightened or diminished response of organisms for survival under stress conditions. Immense changes in DNA methylation level and profile observed for the SL line 6 h after the herbicide application (18.64%) may lead to the deregulation of gene expression, which as a result ends with the plant’s death. In contrast, the change in DNA methylation level in the TL line following herbicide application was very slight (1.13%). Among the DNA sequences identified based on the changes in DNA methylation were transferases, transporter proteins, methyltransferases, hydrolases, transposons, ribosomal proteins, cytochromes, and proteins participating in transcription processes and involved in stress responses.

It appears that natural resistance of crops to herbicides is much more complicated than just a single-trait change (as observed in genetically modified crops) and is based on many mechanisms and several types of regulation of expression of genetic information (epigenetics, small non-coding RNAs, changes in expression and composition of genic products) that together make up the increased fitness of particular varieties.

## Supplementary Information

ESM 1 (DOCX 13 kb)

ESM 2 (DOCX 13 kb)

ESM 3 (XLSX 31 kb)

ESM 4 (XLSX 30 kb)
