# Supplementary material for: Herbicide stress-induced DNA methylation changes in two Zea mays inbred lines differing in Roundup® resistance
Source: J Appl Genet. 2021 Jan 29;62(2):235–48. doi: 10.1007/s13353-021-00609-4 (PMC8032638; doi:10.1007/s13353-021-00609-4)
Supplement: Supplementary file 1 — (DOCX 13 kb) [file 13353_2021_609_MOESM1_ESM.docx]

**Supplementary Table S1.** The test of independence between different methylation patterns (total methylation, full methylation, and hemi-methylation) for control and herbicide stress conditions in TL maize line.

| **TL** | **band type** | **number of loci at 6h post herbicide application** | **number of loci at 7 days post herbicide application** |
| --- | --- | --- | --- |
| control | unmethylated | 330 | 325 |
| control | hemimethylated | 178 | 225 |
| control | fully methylated | 380 | 338 |
| herbicide | unmethylated | 320 | 360 |
| herbicide | hemimethylated | 211 | 178 |
| herbicide | fully methylated | 357 | 350 |
|  |  |  |  |
| χ^2^ |  | 7,81 | 14,23 |
| p value |  | 0,05 | 0,001 |
| df |  | 2 | 2 |

*χ^2^ – chi-square, df – degree of freedom
